# A bibliometric worldview of breast-conserving surgery for breast cancer from 2013 to 2023

**DOI:** 10.3389/fonc.2024.1405351

**Published:** 2024-07-19

**Authors:** Siyang Cao, Yihao Wei, Jing Huang, Yaohang Yue, Aishi Deng, Hui Zeng, Wei Wei

**Affiliations:** ^1^ Department of Breast and Thyroid Surgery, Peking University Shenzhen Hospital, Shenzhen, Guangdong, China; ^2^ National & Local Joint Engineering Research Centre of Orthopaedic Biomaterials, Peking University Shenzhen Hospital, Shenzhen, Guangdong, China; ^3^ Shenzhen Key Laboratory of Orthopaedic Diseases and Biomaterials Research, Peking University Shenzhen Hospital, Shenzhen, Guangdong, China

**Keywords:** global scientific frontiers, breast cancer, breast-conserving surgery, scientometrics, visualization analysis

## Abstract

Over the last decade, significant advancements have been made in breast-conserving surgery (BCS) for breast cancer. However, there is a lack of analytical and descriptive investigations on the trajectory, essential research directions, current research scenario, pivotal investigative focuses, and forthcoming perspectives. The objective of this research is to provide a thorough update on the progress made in BCS for breast cancer over the preceding decade. Retrieved from the Web of Science database, the data span from January 1, 2013, to November 30, 2023. Utilizing a set of advanced analytical instruments, we conducted comprehensive bibliometric and visual analyses. The findings underscore the predominant influence of the USA, representing 35.77% of the overall publications and playing a pivotal role in shaping research within this field. Notable productivity was evident at various institutions, including the Memorial Sloan Kettering Cancer Center, the University of Texas MD Anderson Cancer Center, and the University of Toronto. *Annals of Surgical Oncology* contributed the most publications in this field. An examination of keywords indicated a change in the concentration of research attention, transitioning from molecular subtype, ultrasonography, and intraoperative aspects to SEER, male breast cancer, and adjuvant measures. By offering a comprehensive bibliometric assessment, this study enhances our understanding of BCS for breast cancer. Consequently, this benefits both experienced researchers and newcomers alike, providing prompt access to essential information and fostering the extraction of innovative concepts within this specific field.

## 1 Introduction

Breast cancer stands out as the prevalent form of malignancy affecting women, persistently increasing over the years (1). Worldwide, this disease poses a significant hazard to the physical and mental well-being of women. However, the transformative advancements in primary systemic therapy have revolutionized the approach to managing breast cancer. With a focus on guaranteeing an overall therapeutic effect, the primary consideration and prospective trajectory in breast surgery involve reducing the scope of surgical procedures and improving the quality of life (QoL) for patients (2).

The primary aim of oncologic surgical interventions is cancer removal, entailing the excision of the tumor along with adjacent normal tissue margins. However, an increasing acknowledgment emphasizes the critical significance of aesthetic results in these surgical procedures (3). Patient expectations are on the rise as they comprehend that post-breast cancer surgery deformities are not unavoidable. Favorable aesthetic results have shown a strong association with significant improvements in both patient satisfaction and overall QoL (4, 5). Surgical interventions for breast cancer have witnessed substantial evolution, transitioning from the radical mastectomy pioneered by Halsted in 1894 to the recent establishment and widespread acceptance of breast-conserving therapy as the prevailing standard of care. Breast-conserving surgery (BCS) typically involves lumpectomy or wide local excision. Research has demonstrated that opting for BCS followed by radiotherapy yields comparable outcomes in disease-free and overall survival when compared to mastectomy, solidifying it as the favored approach for early-stage breast cancer (6–9).

While a series of reviews have explored BCS in breast cancer from various perspectives previously (3, 10–20), these assessments frequently lack substantiation through objective visualized data. Instead, they heavily depend on the subjective comprehension of the disciplinary framework by researchers. As a result, a certain level of variability and subjectivity is evident in these evaluations, hindering a comprehensive analysis and establishment of the current state of research. It also poses challenges in identifying research focal points and determining cutting-edge directions. To overcome these constraints, the current investigation utilized scientometric analysis to visually portray the panorama of publications, nations/regions, authors, organizations, keywords, references, fields, and journals within the realm of “BCS for breast cancer” over the last decade. Analyzing the current distribution of research output, acknowledging major contributors, identifying hotspots, assessing current status, and exploring frontiers are the aims of this comprehensive analysis. By establishing such a systematic and comprehensive knowledge base, researchers from various fields will find it easier to navigate the breadth of the domain. Additionally, it acts as a beneficial tool for scholars new to the field, directing them towards intriguing research paths. To our knowledge, there have been no prior bibliometric investigations on this particular subject matter.

## 2 Materials and methods

### 2.1 Data source and retrieval strategy

The Web of Science Core Collection (WoSCC) (https://www.webofscience.com/wos/) facilitates the monitoring of scientific frontiers’ evolution, allowing researchers to comprehensively analyze and understand trends in academic publications (21–24). Serving as a pivotal platform, WoSCC provides bibliometric software for general statistics (23), and its superior accuracy in labeling document types has been demonstrated compared to other databases (25). Within this research, a thorough online exploration was carried out within WoSCC, concentrating on original studies and reviews associated with “BCS for breast cancer”. The investigation encompassed publications spanning from January 1, 2013, to November 30, 2023, employing both Medical Subject Heading terms and free words for data retrieval. The retrieval methodology underwent several revisions, guided by a team of three researchers (YHY, JH, and ASD), aiming to augment sensitivity and precision, as extensively elucidated in the **Supplementary Materials**.

### 2.2 Inclusion and exclusion standards

The inclusion criteria encompassed studies on “BCS for breast cancer”, including original research articles and reviews published in English. Dissertations, case reports, letters, commentaries, editorials, conference abstracts, and studies published under similar or distinct titles in different journals were excluded. Members of the team and peer groups discussed inclusion and exclusion criteria extensively.

### 2.3 Bibliometric visualization and data analysis

Data organization was conducted using Microsoft Excel (Office 365, Microsoft), while co-occurrence analysis was performed using VOSviewer 1.6.18 (Leiden University, Netherlands) and Pajek 64 5.16 (University of Ljubljana, Slovenia). Citespace version 6.2.6R (Chaomei Chen, China) was employed for visual mapping, and Scimago Graphica version 1.0.35 (https://www.graphica.app/, USA) was utilized for graphical analysis. Additionally, specialized graphics were generated using various R packages (R Studio, version 4.2.0), including chorddiag, bibliometrix, complexheatmap (version 2.16.0), and circlize (version 0.4.15).

Chorddiag and Bibliometrix R packages, in conjunction with VOSviewer, were employed to create maps depicting national/regional collaboration and publication analysis charts. VOSviewer, Scimago Graphica, and Pajek were used to conduct co-occurrence analyses covering institutions, journal publications, research fields, and keywords. Information pertaining to countries/regions, institutions, authors, journals, co-citations, and keywords was visualized and mapped using Citespace. Keyword heatmaps were produced using the ComplexHeatmap R package and circlize R package. The temporal variation of keyword popularity was examined using Scimago Graphica.

## 3 Results and discussion

### 3.1 Scientific output

**Figure 1:**
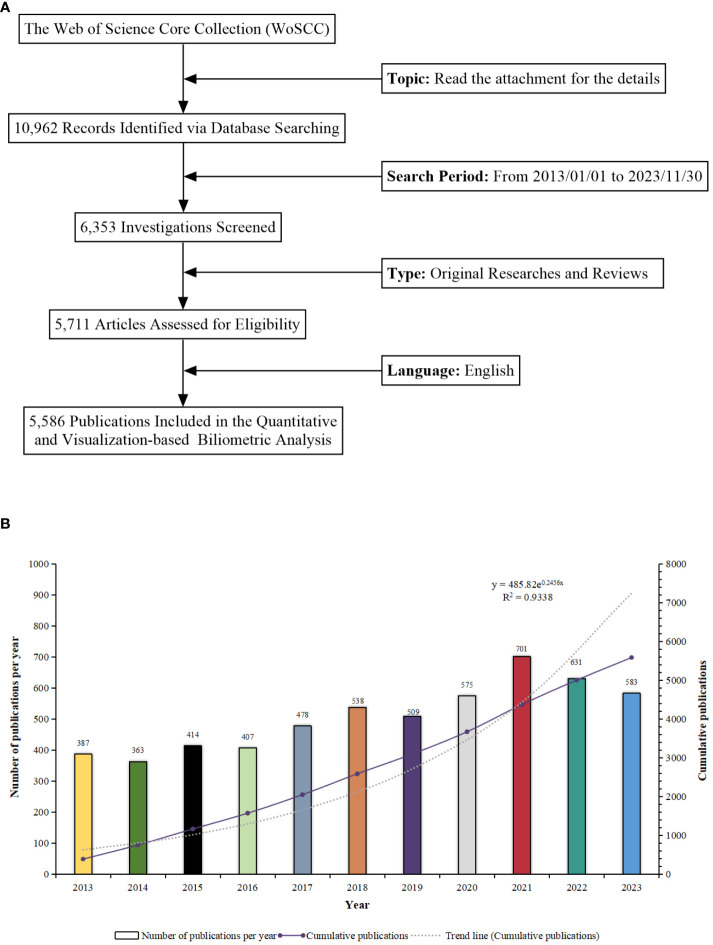
**(A)** Schematic representation of the literature search and selection process. **(B)** Trend analysis of research on “Breast-Conserving Surgery and Breast Cancer” from 2013 to 2023.

The method of retrieving and collecting data is illustrated in **Figure 1A**. The research progress of a study can be indicated by the quantity of scientific reports it produces within a specified period (26–29). From 2013 to 2023, a cumulative total of 5,586 pertinent scientific reports focusing on “BCS for breast cancer” were assembled. This compilation comprised 4,978 original articles and 608 reviews, yielding an average annual publication rate of 558.6. This highlights the substantial attention and interest directed towards this field. Commencing in 2018, the annual tally of relevant publications surpassed 500, reaching its pinnacle at 701 in 2021. The emergence of the COVID-19 pandemic seems to have expedited the output within this domain. The annual trend was accurately depicted using an exponential equation (y = 485.82e^0.2456x^, where x indicates the year and y indicates the number of publications, with R^2^ = 0.9338). This underscores the precision and accuracy applied in the analysis of data, yielding a well-fitted curve (**Figure 1B**). This insight is valuable for researchers, providing a clear overview of the field’s progression and the increasing significance of BCS for breast cancer.

### 3.2 Countries/regions

**Figure 2:**
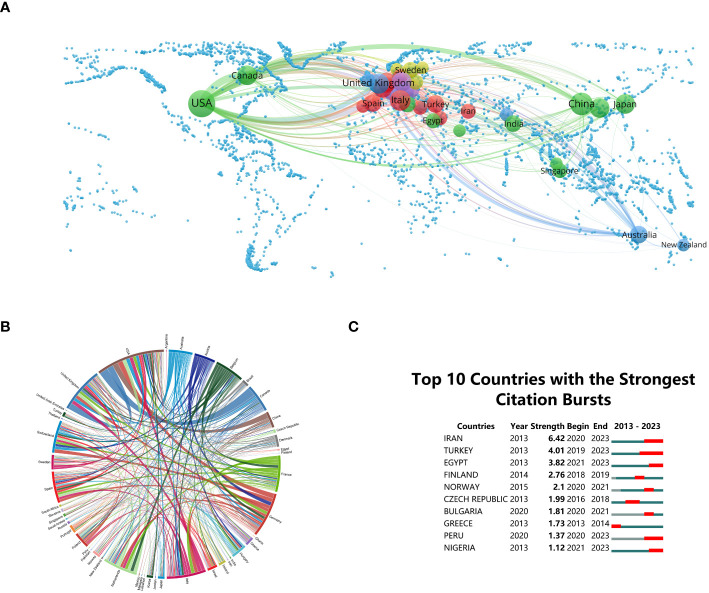
**(A)** Global distribution of “Breast-Conserving Surgery (BCS) and Breast Cancer” research. **(B)** Chord diagrams illustrating international collaborations. **(C)** Research output on “BCS and Breast Cancer” from the top 10 countries (highlighted in red, signifying increases in document production).

Research on “BCS for breast cancer” involves 113 countries/regions globally. Establishing a minimum publication count of twenty from each country/region, the construction of national collaboration networks is depicted in **Figures 2A, B**. This provides a tangible representation of the prominence of each country or region within the domain, offering valuable insights for strategic collaborations and knowledge sharing (30). Notably, the USA leads with 1,998 publications, constituting 35.77% of the total research output, emphasizing its pivotal role in advancing knowledge in BCS. Subsequently, China and Italy make noteworthy contributions, accounting for 12.42% (694 publications) and 6.53% (365 publications), respectively, in the global research on “BCS for breast cancer”, highlighting the global nature of this research and encouraging collaboration and the exchange of expertise on an international scale.

The peripheral curve segments in the chord diagram visually represent countries and regions. The length of each segment corresponds to the publication volume of the respective country or region (31). Connectivity among nations reflects their levels of collaborative engagement. In terms of international collaboration, the USA exhibits the highest frequency, primarily partnering with Canada (link strength = 89) and the United Kingdom (link strength = 62) (**Figure 2B**). This information holds immense value for researchers seeking potential collaborators and industry practitioners aiming to stay informed about international collaborations that may impact clinical practices (32).

Identifying publications that have experienced substantial increases in citations over a designated time frame is crucial, and this is achieved through the recognition of citation bursts (33). **Figure 2C** illustrates the citation bursts for the top 10 countries, with the magnitude of each burst represented by the red line. Noteworthy is the significant surge in publication citations experienced by Iran (strength = 6.42) between 2019 and 2023, closely followed by Turkey (strength = 4.01) and Egypt (strength = 3.82). This information provides researchers with the opportunity to delve into emerging topics, while industry practitioners can harness these insights to foresee and adjust to evolving trends in BCS.

### 3.3 Institutions

**Figure 3:**
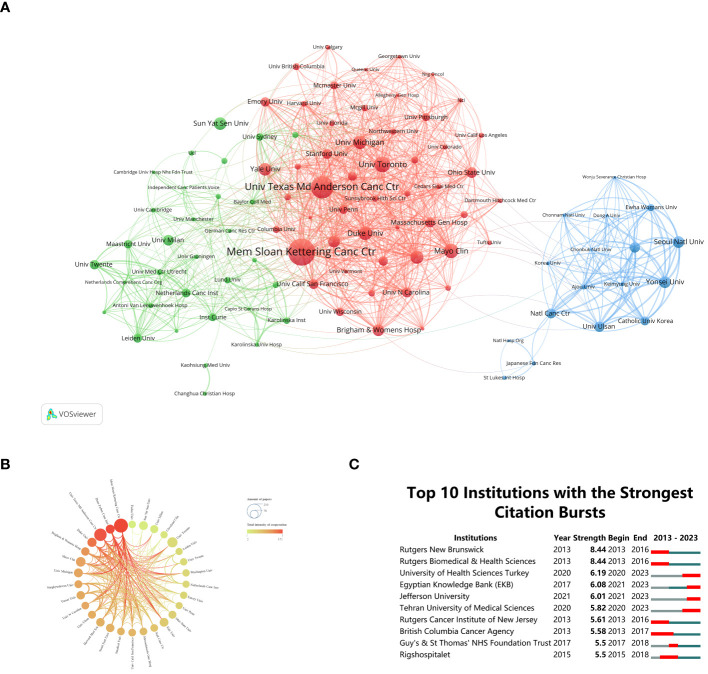
**(A)** Clustering networks of relevant research institutions. **(B)** Diagram of institutional cooperation intensity. **(C)** Citation bursts at the top 10 institutions (red bars represent burst periods for institutions).

By analyzing the dynamic collaborative network among institutions, researchers can gain insights into the vibrant research ecosystem in the “BCS for breast cancer” domain, enabling them to strategically plan collaborations and knowledge sharing. Research institution cooperation relationship maps and clustering maps were generated using a minimum publication threshold of fifty documents per institution (**Figures 3A, B**). Distinct clustering information is represented by different colored regions. The intensity of collaboration is depicted by the thickness of connections between circles, while the size of each circle corresponds positively to the number of documents published by each organization. Over the past decade, the global research landscape in “BCS for breast cancer” has witnessed substantial growth, involving over 6,012 entities. The most prolific institution was Memorial Sloan Kettering Cancer Center (n = 227, 3.4%), with the University of Texas MD Anderson Cancer Center (n = 152, 2.72%) and the University of Toronto (n = 101, 1.81%) following closely. These institutions become potential collaborators for future research endeavors, presenting opportunities for collaborative projects and knowledge exchange. Regarding collaboration between institutions, Memorial Sloan Kettering Cancer Center took a prominent role, showcasing a strong commitment to partnering with other establishments. This commitment is evident through substantial affiliations between Memorial Sloan Kettering Cancer Center and nearly all notable scholarly organizations.

Furthermore, insights into institutions experiencing citation bursts are crucial for researchers and industry practitioners alike. Grasping the lasting influence and adaptability of research initiatives across time enables a thorough assessment of an institution’s research undertakings (33). Through CiteSpace analysis (**Figure 3C**), this investigation pinpointed institutions experiencing notable citation surges. Rutgers New Brunswick and Rutgers Biomedical & Health Sciences tied for first place, both showing a burst from 2013 to 2016 (strength = 8.44). The British Columbia Cancer Agency underwent the lengthiest bursts in citations between 2013 and 2017. Regrettably, this pattern has not persisted over the past six years. In contrast, the University of Health Sciences Turkey, Egyptian Knowledge Bank, Jefferson University, and Tehran University of Medical Sciences experienced a citation surge deferred from 2020 to 2023. Among these institutions, this pattern indicates a shift in focus and postponement of research.

In summary, these findings empower researchers to strategically navigate collaborations, leverage the contributions of top-performing institutions, and stay informed about emerging trends in research output and focus areas within the “BCS for breast cancer” domain.

### 3.4 Authors

**Figure 4:**
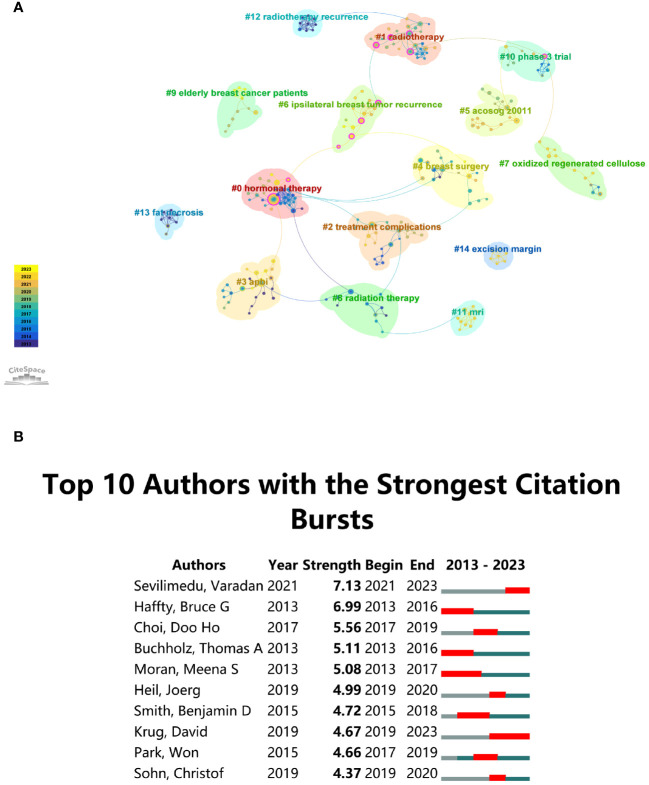
**(A)** Author cluster analysis. **(B)** Top 10 authors with significant citation bursts in “Breast-Conserving Surgery for Breast Cancer” publications.

The Modularity (*Q* value) and Mean Silhouette (*S* value) of CiteSpace are used to evaluate the integrity of the network and the clarity of clustering. Strong clustering is indicated by a *Q* value exceeding 0.3, while a distinct and reasonable clustering is suggested by an *S* value over 0.5 (34). According to the investigation, keyword clusters are highly significant and well-defined with a cluster modularity value (*Q*) of 0.9232 and a mean silhouette value (*S*) of 0.9799. This provides researchers with a valuable tool for navigating and comprehending the intricate landscape of research topics in the domain. In **Figure 4A**, 15 author groups are delineated and annotated with corresponding keywords, encompassing: #0 hormonal therapy, #1 radiotherapy, #2 treatment complications, #3 APBI, #4 breast surgery, #5 ACOSOG Z0011, #6 ipsilateral breast tumor recurrence, #7 oxidized regenerated cellulose, #8 radiation therapy, #9 elderly breast cancer patients, #10 phase 3 trial, #11 MRI, #12 radiotherapy recurrence, #13 fat necrosis, #14 excision margin. This is particularly beneficial for researchers seeking collaboration opportunities, offering a comprehensive overview of key thematic clusters within “BCS for breast cancer”.

Citation burst analysis is a crucial metric, reflecting the frequency with which an author receives citations in a particular research domain over a specified timeframe (35, 36). **Figure 4B** displays the top ten authors within the “BCS for breast cancer” domain who have obtained the highest number of citations. At the forefront of the list is Varadan Sevilimedu, demonstrating a burst strength of 7.13, with close followers being Bruce Haffty and Choi Doo-ho. Significantly, there has been a noteworthy upswing in publication output over the past four years for authors Varadan Sevilimedu and David Krug, indicating their particular emphasis on research within this specific field.

For industry practitioners, understanding the citation bursts and identifying influential authors is crucial for staying abreast of the latest developments and trends in “BCS for breast cancer”. By recognizing researchers with the highest citation counts, practitioners can identify experts to consult for clinical insights and innovation. Overall, these findings provide a comprehensive and actionable resource for both researchers and industry practitioners, fostering collaboration and informed decision-making in the dynamic landscape of “BCS for breast cancer” research.

### 3.5 Journals and related fields

**Figure 5:**
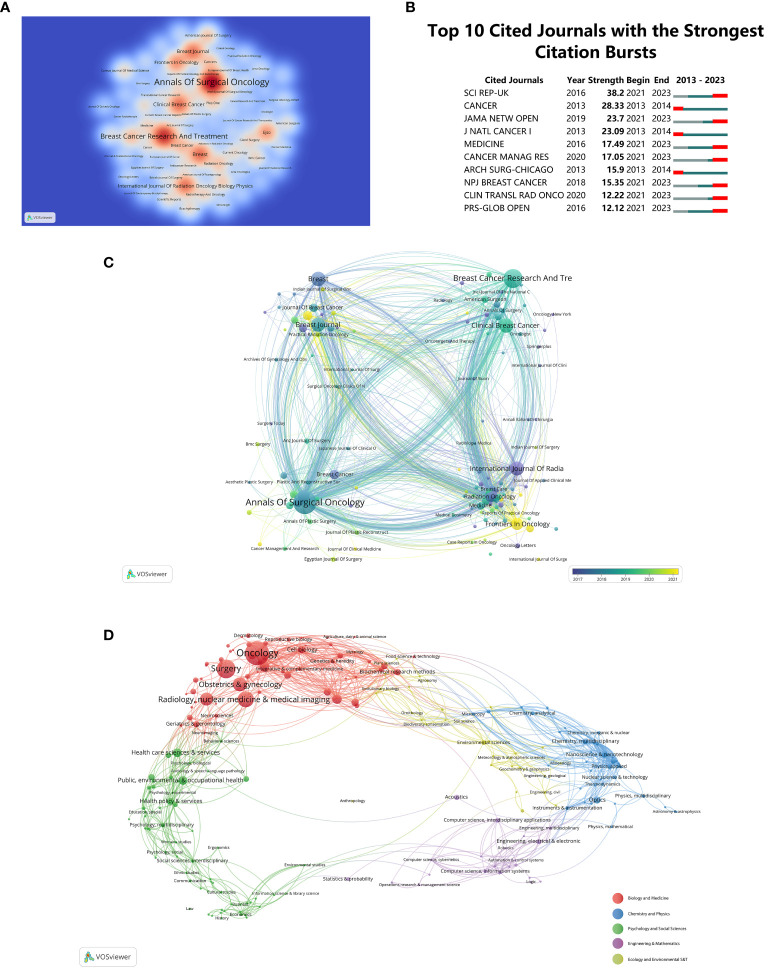
**(A)** Density visualization map of journal citations. **(B)** Top 10 Journals with the strongest citation bursts. **(C)** Journal distribution based on the average publication year (blue: earlier, yellow: later). **(D)** Analyses of research subject areas.

Visualized data on journal publications reveals that 800 journals have published articles on “BCS for breast cancer”. **Figure 5A** illustrates the thermodynamic chart showcasing the distribution of documents among journals, with a minimum threshold of fifteen documents per journal. The color intensity on the chart corresponds to the quantity of published journal papers. Leading in the number of published documents (n = 347, 6.21%) is the journal ‘*Annals of Surgical Oncology*’, followed by ‘*Breast Cancer Research and Treatment*’ (n = 246, 4.4%), and ‘*Breast*’ (n = 152, 2.72%). A comprehensive grasp of the publication landscape in this domain will aid researchers in selecting suitable journals for their work, thereby ensuring widespread dissemination of their research findings. **Figure 5B** presents a compilation of the top 10 journals showcasing the most notable citation bursts for articles concerning “BCS for breast cancer”. This insight aids in prioritizing citations and references, enabling researchers to align their work with influential publications in the “BCS for breast cancer” domain.

Diverse journals are differentiated by color, emphasizing their average year of inception in **Figure 5C**. Frequency of occurrence is denoted by circles and labels, while circle color signifies the mean publication year. Manifestly, ‘*World Journal of Clinical Cases*’ and ‘*Journal of Personalized Medicine*’ are presently burgeoning journals, as evidenced by their representation in yellow. VOSviewer software visually categorized 5,586 articles into five major fields. Clustering is depicted in **Figure 5D**, utilizing differently colored spheres for distinct domains. Results indicate a concentration of research in “Biology and Medicine”, with a notable proportion in “Oncology”, “Surgery”, “Radiology”, and “Nuclear Medicine & Medical Imaging”.

### 3.6 Co-cited references

**Figure 6:**
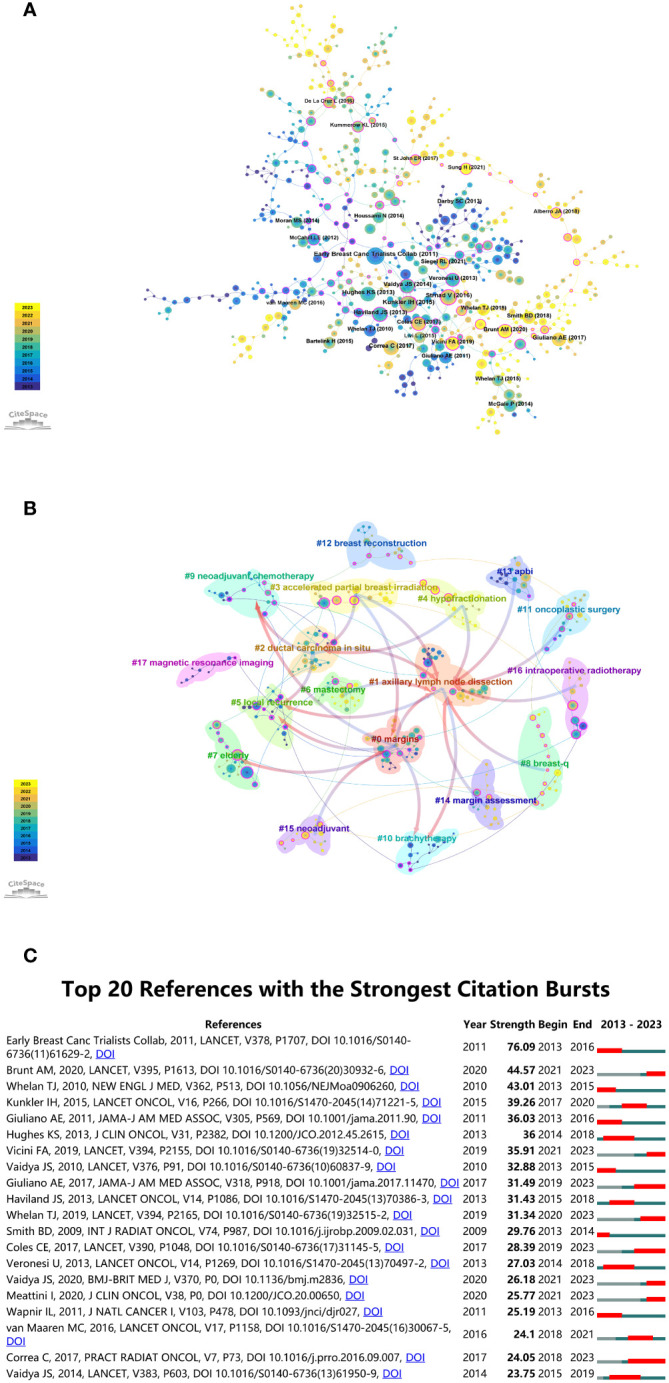
**(A)** Co-citation analysis chart for “Breast-Conserving Surgery for Breast Cancer”. **(B)** Co-cited literature network map. **(C)** Top 20 references with the highest citation bursts.

Researchers can gain insights into influential literature that has significantly contributed to the advancement of knowledge in the domain by pinpointing key works with the highest co-citation frequencies. Using CiteSpace, **Figure 6A** illustrates the co-citation connections within literature related to “BCS for breast cancer” from January 1, 2013 to November 30, 2023. The sizes of the spheres, aggregated across annual rings, directly reflect the co-citation frequencies. In the color spectrum, purple indicates older citations, while yellow indicates more recent citations. Citations over the specified years are represented by overlapping colors in the spheres. Co-citation relationships between different literary works are depicted by the connecting lines between spheres. The magenta nodes, with a centrality greater than 0.1, represent important nodes in the network. The review titled ‘Effect of radiotherapy after breast-conserving surgery on 10-year recurrence and 15-year breast cancer death: meta-analysis of individual patient data for 10,801 women in 17 randomized trials’, authored by the Early Breast Cancer Trialists’ Collaborative Group et al. and published in *The Lancet*, distinguishes itself as one of the most often cited documents, boasting the highest co-citation count (n = 203), indicating its pivotal role in shaping scholarly discourse (37). The 2015 *Lancet Oncology* paper ‘Breast-conserving surgery with or without irradiation in women aged 65 years or older with early breast cancer (PRIME II): a randomized controlled trial’ by Ian H Kunkler et al. follows with 175 co-citations (38).

CiteSpace employs metrics like Modularity (*Q* value) and Mean Silhouette (*S* value) to evaluate network structure and clustering clarity. A *Q* value surpassing 0.3 indicates significant clustering, while an *S* value exceeding 0.5 indicates clear and effective clustering. Analysis yielded computed values of *Q* = 0.8748 and *S* = 0.9551, affirming the existence of robust clustering structures. Citation clustering is proven to be reliable based on this result. The analysis revealed 18 unique clusters, labeled as #0 margins, #1 axillary lymph node dissection, #2 ductal carcinoma *in situ*, #3 accelerated partial breast irradiation, #4 hypofractionation, #5 local recurrence, #6 mastectomy, #7 elderly, #8 breast-q, #9 neoadjuvant chemotherapy, #10 brachytherapy, #11 oncoplastic surgery, #12 breast reconstruction, #13 APBI, #14 margin assessment, #15 neoadjuvant, #16 intraoperative radiotherapy, #17 magnetic resonance imaging, as illustrated in **Figure 6B**. This data enables researchers to align their work with recognized research clusters, thereby enhancing the chances of acknowledgment and citations from peers and experts.

Utilizing CiteSpace’s analytical features, we identified citation bursts, offering insights into research areas that have garnered substantial scholarly interest within the domain of “BCS for breast cancer”. The identification of citation bursts, as presented in **Figure 6C**, provides a temporal perspective on the scholarly impact of studies in the “BCS for breast cancer” field. As of 2013, there has been a notable increase in citations in the field, with various co-citation references accumulating significant citations over the years. As a result of this trend, breast cancer research continues to be of enduring significance. Among these references, 30% (6 out of 20) showed citation bursts in 2013, making it the year with the highest frequency. In second place, 2021 accounted for 20% (4 out of 20 bursts). The investigation with the highest citation burst (strength = 76.09) was titled ‘Effect of radiotherapy after breast-conserving surgery on 10-year recurrence and 15-year breast cancer death: meta-analysis of individual patient data for 10,801 women in 17 randomized trials’, originally published in *The Lancet* (37). The influence was subsequently echoed by the contributions of Adrian Murray Brunt and colleagues (39), and Timothy J Whelan and collaborators (40). This temporal information equips researchers to stay abreast of emerging trends, allowing them to channel their efforts toward high-impact areas. For industry practitioners, an understanding of these scholarly dynamics is equally crucial. Apart from guiding strategic decisions, this data facilitates the identification of research partnerships and investment opportunities aligned with the most cited and impactful studies.

### 3.7 Keywords

**Figure 7:**
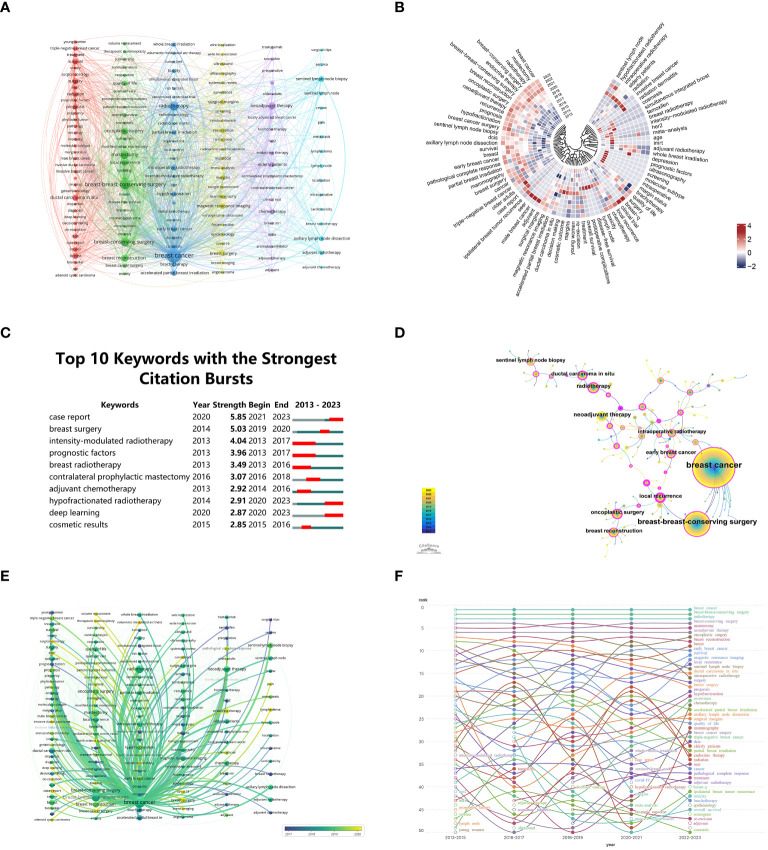
**(A)** Keywords clustering visualization. **(B)** Annual heatmap from 2013 to 2023. **(C)** Top 10 Keywords with significant citation bursts. **(D)** Co-occurrence analysis chart of keyword frequencies. **(E)** Temporal frequency spectrum of breast cancer-related keywords. **(F)** Keyword heat trend graph.

Academic articles rely heavily on keywords to reflect the existing knowledge base and guide advancements within a particular field. A visual map (**Figure 7A**) was generated from co-occurrence cluster analysis of keywords, where nodes are represented by circles. The size of circles corresponds directly to keyword frequency, while the strength of relationships among these keywords is indicated by the thickness of connecting lines. The nodes were categorized by color, with each cluster representing a distinct research path. Five clusters were successfully identified. The resulting co-occurrence cluster analysis and visual map offer a structured representation of the current knowledge foundation and the interrelationships among keywords. This visual representation, featuring nodes and clusters, provides researchers with a clear understanding of diverse research directions within the academic domain.

In **Figure 7B**, the annual popularity of keywords (calculated as the number of citations in the year divided by the total citations in the year) from 2013 to 2023 is depicted. Notably, keywords such as molecular subtype, ultrasonography, and intraoperative have demonstrated comparatively modest annual popularity in recent years. Conversely, keywords such as SEER, male breast cancer, and adjuvant have exhibited relatively high annual popularity, suggesting these terms represent emerging frontier areas. Researchers can strategically align their work with these trends, ensuring their contributions remain relevant and impactful.

By detecting keyword bursts, especially those experiencing notable increases in citations, heightened scholarly focus can be identified (**Figure 7C**). The initial surge in citations of 30% of the keywords (3 out of 10) occurred in 2013, closely followed by the surge of 20% in 2020 (2 out of 10). Approximately 30% of these keywords have sustained high citation rates in the past three years, suggesting ongoing and increasing interest. The keyword ‘case report’ experienced a notable burst with a burstiness value of 5.85, followed by ‘breast surgery’ and ‘intensity-modulated radiotherapy’ with burstiness values of 5.03 and 4.04 respectively. As a result of these insights, researchers can prioritize their efforts in areas that are currently receiving significant attention from the research community. In **Figure 7D**, CiteSpace is used to analyze the simultaneous appearance of keywords related to “BCS for breast cancer” from January 1, 2013, to November 30, 2023. This representation unveils the interrelated nature of keywords throughout this timeframe. The sizes of superimposed circles, determined by aggregating the sizes of circles associated with yearly rings, are commensurate with the frequency of keyword occurrences. Purple denotes keywords that surfaced relatively early, whereas yellow indicates those that emerged later. The occurrence of overlapping colors on the chart signifies the citation frequency of the respective keywords in the corresponding years. A connecting line between circles represents co-citation relationships, while a magenta node denotes a pivotal node with centrality exceeding 0.1. Alongside the search terms “breast cancer” and “breast-conserving surgery”, the most commonly co-occurring keywords include “neoadjuvant therapy” and “radiotherapy”.

**Figure 7E** depicts a chronological keyword spectrum related to “BCS for breast cancer”, where line thickness correlates with the intensity of association. Keywords in blue signify earlier appearances, indicating their foundational status in the field, while those in yellow denote more recent developments, suggesting emerging research directions. This spectrum effectively illustrates the evolution of research themes, key concepts, and the interrelationships among various ideas over time. Consequently, it provides a nuanced understanding of the knowledge landscape and research endeavors within this domain, enabling scholars to gain insights into the historical development of key concepts and identify emerging research directions. Examining articles published between 2013 and 2023, **Figure 7F** portrays the evolution of keywords over the last decade. A circle with an open center indicates the emergence of a keyword over a nearly ten-year period, while a circle with a filled center indicates its culmination.

From **Figure 7F**, it is evident that numerous topics have consistently remained hotspots in “BCS for breast cancer”. Due to space limitations, we focus on the following key topics, based on our clinical experience:

#### 3.7.1 The survival of elderly patients receiving BCS

In recent years, the incidence of breast cancer in the elderly has increased, with over 30% of patients being over 79 years old (41). Studies show that the acceptance rate of radiotherapy decreases by 13.1% for each additional year of age (42). Margin involvement, grading, and lymphovascular invasion are risk factors for local breast cancer recurrence. These factors are mainly derived from clinical trial data of women under 70 and cannot accurately predict recurrence in elderly patients (43). Current clinical evidence on the safety of omitting radiotherapy in elderly patients with early-stage breast cancer is limited. PRIME II is a phase III multicenter clinical trial comparing the risk of local recurrence with or without radiotherapy in women aged 65 and above with early-stage breast cancer who underwent BCS (38). The study included 1,326 elderly breast cancer patients who underwent BCS and were followed for 5 years. The results showed 5-year ipsilateral breast recurrence rates of 1.3% for those who received radiotherapy and 4.1% for those who did not. There were no differences between the two groups in regional recurrence, distant metastasis, contralateral breast cancer, or new primary breast cancer. Radiotherapy lowered ipsilateral breast recurrence rates, but there was no significant difference in the 5-year overall survival rate between the groups. Another meta-analysis further confirmed this conclusion (13). This analysis included 10 prospective studies with 5,271 elderly breast cancer patients aged 50 and above who underwent BCS, evaluating survival outcomes of radiotherapy plus endocrine therapy versus endocrine therapy alone. Results showed that compared to endocrine therapy alone, radiotherapy reduced the 5-year ipsilateral breast recurrence rate but did not affect the 5-year overall survival.

For elderly patients with early-stage breast cancer, omitting radiotherapy does not affect survival rates. Thus, can endocrine therapy also be omitted? A study included 888 estrogen receptor-positive/human epidermal growth factor receptor 2-negative T_1_N_0_ breast cancer patients over 65 who underwent BCS (44). They were divided into four groups based on adjuvant therapy: radiation monotherapy, adjuvant hormonal monotherapy, combined radiation and hormonal therapy, or neither. Results showed five-year locoregional recurrence rates of 11% for no adjuvant treatment, 3% for adjuvant hormonal monotherapy, 4% for radiation monotherapy, and 1% for combined radiation and hormonal therapy. Locoregional recurrence rates differed significantly between the groups. Distant recurrence and breast cancer-specific survival rates did not differ significantly between groups. Therefore, although radiotherapy and/or endocrine therapy reduced local recurrence rates, they did not significantly affect overall survival. For hormone receptor-positive elderly breast cancer patients, completely omitting endocrine therapy and radiotherapy is not feasible. Considering treatment toxicity and side effects on quality of life, further prospective trials are needed to explore how elderly patients should choose radiotherapy and/or hormone therapy after BCS.

#### 3.7.2 BCS after neoadjuvant chemotherapy (NAC)

Although BCS after NAC has become an important treatment method, it remains highly controversial. Recently, the most contentious issue is whether BCS following NAC-induced tumor downstaging increases the risk of local recurrence in breast cancer patients. A meta-analysis published in 2018 included 10 randomized trials with 4,756 patients treated between 1983 and 2002. This analysis showed that, at a median follow-up of 9 years, patients who underwent BCS after NAC had a significantly higher local recurrence rate compared to those who received surgery followed by chemotherapy (21.4% vs. 15.9%) (45). The authors hypothesized that the increased local recurrence rate could be attributed to patients who were originally unsuitable for BCS but underwent BCS after tumor downstaging. The 1998 NSABP B-18 trial made subgroup comparisons to address this hypothesis (46). The local recurrence rate was 15.9% (11/69) for patients who underwent BCS after tumor downstaging, compared to 9.9% (43/434) for patients originally suitable for BCS. After adjusting for patient age and initial tumor size, the difference was no longer statistically significant.

A clinical trial published in 2022 also produced similar results (47). This study retrospectively tracked 685 clinical stage T_1_-T_3_ breast cancer patients from 2014 to 2018, dividing them into three groups: those originally suitable for and ultimately undergoing BCS, those unsuitable for BCS who underwent BCS after downstaging, and those unsuitable for BCS who, despite downstaging, opted for mastectomy. 92% of patients received the doxorubicin + cyclophosphamide + taxane NAC regimen, and 99% of BCS patients received adjuvant radiation. Researchers compared the clinical and pathological characteristics and local recurrence rates of the patients. At a median follow-up of 35 months, the 4-year local recurrence-free survival rates were similar across the three groups. This study suggests that even patients initially unsuitable for BCS did not show significant improvement in local recurrence rates after opting for mastectomy. However, this study has several limitations, including its retrospective nature, insufficient follow-up time, and the absence of a control group of patients who did not receive NAC. Therefore, more high-quality clinical evidence is needed to guide clinical decision-making.

#### 3.7.3 BCS for patients with ipsilateral breast cancer recurrence

Ipsilateral breast recurrence is the most common form of recurrence after BCS (48). Many studies indicate that ipsilateral breast recurrence independently predicts distant metastatic disease or cancer-specific mortality (49, 50). Nevertheless, treating ipsilateral breast recurrence remains controversial. Due to severe complications from secondary radiotherapy, such as pulmonary fibrosis, ischemic heart disease, and rib fractures (51), the National Comprehensive Cancer Network currently recommends mastectomy for ipsilateral breast recurrence (52). However, some studies suggest that patients with ipsilateral breast recurrence may still benefit from BCS alone without additional radiotherapy. A study of 121 patients diagnosed between 1987 and 2014 with pT_0-2_N_0-3_ who underwent BCS and radiotherapy was conducted (53). After ipsilateral breast recurrence, 47 patients had another BCS, and 74 patients underwent mastectomy. Results showed that over an average follow-up of 14 years, 8 BCS patients (17%) and 10 mastectomy patients (13.5%, *P*=0.22) experienced local recurrence. During the 14-year follow-up, the survival rate was 95.8% for patients with repeat BCS compared to 87% for those who had mastectomy (*P*=0.012). Although repeat BCS after ipsilateral breast recurrence has a higher recurrence rate, it does not affect long-term survival.

Another study examined the feasibility of re-irradiation for ipsilateral breast recurrence patients (54). This study included 34 patients with ipsilateral breast recurrence after BCS and radiotherapy, who were re-irradiated after recurrence. The median follow-up was 23.5 months, and the average recurrence interval was 9.8 years. No patients experienced toxicity greater than grade 3; the main acute toxicity was radiation dermatitis. Therefore, for patients who tolerate radiotherapy, re-irradiation after ipsilateral breast recurrence is a viable option. The third study suggested that a second BCS combined with partial breast re-irradiation is an effective alternative to mastectomy (55). This study included 58 patients with ipsilateral breast recurrence after BCS and radiotherapy, all of whom underwent a second BCS and partial breast re-irradiation, with a median follow-up of 5.5 years. The results showed a 3-year cumulative recurrence rate of 3.4% and a 5-year cumulative recurrence rate of 5.2%. The distant metastasis–free survival and overall survival rates were both 95%, and all adverse events were below grade 3. Therefore, second BCS and partial breast re-irradiation are feasible and effective for patients with ipsilateral breast recurrence. These results are consistent with previous studies, which had smaller sample sizes and selection bias, leading to lower overall evidence quality (56–58). However, other studies have shown opposite results, suggesting better survival outcomes for patients undergoing mastectomy compared to those undergoing BCS (59–61).

To date, no prospective studies have investigated second BCS combined with re-irradiation for patients with ipsilateral breast recurrence. Existing evidence is still insufficient to support second BCS combined with re-irradiation as the preferred option for these patients.

#### 3.7.4 QoL after BCS

The average lifespan of breast cancer patients is increasing, and there is growing attention on postoperative QoL (62). Understanding postoperative patients’ subjective symptoms and QoL aids in shared decision-making for surgical management. Early studies suggested no significant difference in QoL between mastectomy and BCS (63, 64), but these were limited by small sample sizes and short follow-up periods. A 2019 cohort study evaluated subjective symptoms in 13,865 stage I-II breast cancer patients (11,497 in the BCS + radiotherapy group and 2,368 in the mastectomy group) (65). Researchers conducted a 12-month follow-up, using the Edmonton Symptom Assessment System to evaluate nine symptoms: pain, fatigue, drowsiness, nausea, loss of appetite, shortness of breath, depression, anxiety, and overall well-being. Results showed that both groups reported similar symptom severity within 5 months post-surgery. Over time, mastectomy patients had significantly higher incidences of moderate-to-severe depression (*P*<0.05), lack of appetite (*P*<0.05), and shortness of breath (*P*<0.05) compared to BCS + radiotherapy patients.

The European Organization for Research and Treatment of Cancer developed the Breast Cancer-Specific Quality-of-Life Questionnaire in 1996 to assess postoperative QoL, including body image, sexual function, systemic treatment side effects, breast symptoms, arm symptoms, and distress from hair loss (66). A meta-analysis using this scale compared postoperative QoL in breast cancer patients undergoing mastectomy and BCS, including six studies (67). Results indicated that BCS patients had significantly better QoL in body image (*P*=0.003), future perspective (*P*=0.025), and systemic therapy side effects (*P*=0.020) compared to mastectomy patients. A 2021 multicenter cross-sectional study included 560 early breast cancer patients aged 40 or younger, with a median follow-up of 5.8 years (68). The study showed that compared to BCS patients, mastectomy patients had significantly lower scores in breast satisfaction, psychosocial health, and sexual health. In summary, within a few years post-surgery, BCS patients had better subjective symptoms and QoL compared to mastectomy patients. However, studies on the long-term impact of BCS on patients’ QoL require longer follow-up.

### 3.8 Possible individual-level and demographic factors affecting surgical treatment options for BCS

For many individuals with nonmetastatic breast cancer, BCS is a safe oncological alternative to mastectomy. Compared to mastectomy, BCS causes less breast deformity while maintaining similar rates of local recurrence and long-term survival (69–72). Additionally, BCS reduces the need for reconstructive surgery and lowers the incidence of lymphedema, thereby enhancing QoL (73). Multiple studies have explored factors influencing patient preferences. Patients might avoid BCS due to cultural norms, a desire to avoid future procedures, anxiety, and fear of recurrence or abnormal physical exam results (74–77). Conversely, aesthetic benefits, especially when combined with modern reconstructive techniques, may lead patients to prefer BCS (78, 79). Studies show that Black patients are less likely to receive timely adjuvant radiation therapy after BCS, with their survival outcomes more closely tied to tumor size than those of White patients (80–82). Data from the Surveillance, Epidemiology, and End Results database indicate that women of lower socioeconomic status are less likely to receive sentinel lymph node biopsy and radiation after BCS compared to those of higher socioeconomic status (83). Recognizing that limited access and structural barriers to treatment may disproportionately hinder certain groups of women from receiving BCS based on various demographic factors, such as race, ethnicity, education, rural or urban residence, socioeconomic status, insurance status, marital status, body mass index, and insurance coverage, is crucial (84–89).

For policymakers, existing issues highlight the need to address significant inequities in access to BCS and associated treatments. This involves ensuring that marginalized groups receive the same level of care and follow-up as more privileged ones. To bridge these gaps, policymakers must develop and implement policies that make BCS more accessible and affordable, possibly through subsidies or insurance reforms. Educational campaigns in less developed areas that target cultural norms and misconceptions about BCS could help increase its acceptance and reduce psychological barriers for patients. Industry practitioners need enhanced training for surgeons and oncologists in BCS techniques and postoperative care to ensure high treatment standards. Furthermore, practitioners should develop and disseminate educational materials that clearly explain the benefits and risks of BCS, aiding patients in making informed decisions.

### 3.9 Strengths and limitations

In contrast to prior inquiries, which primarily leaned on meta-analyses or narrative reviews, the employment of bibliometric tools in this research offered a more transparent depiction of research emphases and trajectories across diverse dimensions (29, 31, 33, 90, 91). This study marks the inaugural effort in the last decade to perform bibliometric analysis for delineating and delineating “BCS for breast cancer” knowledge landscapes, furnishing a thorough and impartial benchmark for forthcoming progressions, notwithstanding the presence of certain unavoidable constraints.

Several limitations were encountered in this investigation. 1) Because of the inherent constraints of CiteSpace, publications were solely extracted from WoSCC, leading to an inevitable selection bias. 2) The citation count, serving as a measure of a paper’s impact, is vulnerable to various confounding factors that might impinge on its accuracy. 3) The credibility of the study might have been compromised due to the extensive volume of papers, which rendered it impractical to thoroughly analyze each paper and subfields. 4) Bibliometric techniques, as suggested by prior bibliometric studies, heavily rely on natural language processing, which has exhibited potential bias (90, 92). 5) The inclusion of only English documents could introduce publication bias. 6) The incompleteness of the literature collection might result in the omission of newly published literature and certain keywords from the statistical analysis during the literature retrieval process.

## 4 Conclusion

Conducted through bibliometric analysis, this research delves into the realm of “BCS for breast cancer”, shedding light on facets such as global collaboration, publication patterns, and pivotal research themes. These insights equip the scientific realm to pinpoint nascent ideas and frontiers poised to shape the trajectory of breast cancer BCS exploration. Advancement in this domain hinges upon researchers not only staying abreast of these dynamics but also harnessing pre-existing wisdom.

## Data availability statement

The raw data supporting the conclusions of this article will be made available by the authors, without undue reservation.

## Ethics statement

As the data utilized in this study originated from publicly accessible sources and did not involve any interaction with human subjects, the need for ethical approval or informed consent was deemed unnecessary.

## Author contributions

SC: Writing – original draft, Formal analysis, Conceptualization. YW: Writing – original draft, Investigation, Data curation. JH: Writing – original draft, Investigation, Data curation. YY: Writing – review & editing, Software, Methodology. AD: Writing – review & editing, Software, Methodology. HZ: Writing – review & editing, Supervision, Project administration, Funding acquisition. WW: Writing – review & editing, Supervision, Project administration, Funding acquisition.
