# Supplementary material for: A bibliometric worldview of breast-conserving surgery for breast cancer from 2013 to 2023
Source: Front Oncol. 2024 Jul 19;14:1405351. doi: 10.3389/fonc.2024.1405351 (PMC11294094; doi:10.3389/fonc.2024.1405351)
Supplement: Supplementary file 1 [file Table_1.docx]

Supplementary Material

**A Bibliometric Worldview of Breast-Conserving Surgery for Breast Cancer from 2013 to 2023**

**Siyang Cao^1, 2, 3,^** **^†^, Yihao Wei^2, 3, †^, Jing Huang^1, †^, Yaohang Yue^2, 3^, Aishi Deng^1^, Hui Zeng^2, 3, *^, Wei Wei^1, *^**

1 Department of Breast and Thyroid Surgery, Peking University Shenzhen Hospital, Shenzhen, Guangdong, People's Republic of China.

2 National & Local Joint Engineering Research Centre of Orthopaedic Biomaterials, Peking University Shenzhen Hospital, Shenzhen, Guangdong, People's Republic of China.

3 Shenzhen Key Laboratory of Orthopaedic Diseases and Biomaterials Research, Peking University Shenzhen Hospital, Shenzhen, Guangdong, People's Republic of China.

***Correspondence authors:**

Hui Zeng (zenghui@pkuszh.com) and Wei Wei (rxwei1123@163.com).

# *Present Address:

# No. 1120 Lianhua Road, Futian District, Shenzhen, Guangdong Province, People's Republic of China.

**†**Siyang Cao, Yihao Wei, and Jing Huang contributed equally to this work and share the first authorship.

*Hui Zeng and Wei Wei contributed equally to this work and share the last authorship.

# Search strategy

| **Search** | **Query** |
| --- | --- |
| #1 | TS=("Breast Neoplasms" or "Breast Neoplasm" or "Neoplasm, Breast" or "Breast Tumors" or "Breast Tumor" or "Tumor, Breast" or "Tumors, Breast" or "Neoplasms, Breast" or "Breast Cancer" or "Cancer, Breast" or "Mammary Cancer" or "Cancer, Mammary" or "Cancers, Mammary" or "Mammary Cancers" or "Malignant Neoplasm of Breast" or "Breast Malignant Neoplasm" or "Breast Malignant Neoplasms" or "Malignant Tumor of Breast" or "Breast Malignant Tumor" or "Breast Malignant Tumors" or "Cancer of Breast" or "Cancer of the Breast" or "Mammary Carcinoma, Human" or "Carcinoma, Human Mammary" or "Carcinomas, Human Mammary" or "Human Mammary Carcinomas" or "Mammary Carcinomas, Human" or "Human Mammary Carcinoma" or "Mammary Neoplasms, Human" or "Human Mammary Neoplasm" or "Human Mammary Neoplasms" or "Neoplasm, Human Mammary" or "Neoplasms, Human Mammary" or "Mammary Neoplasm, Human" or "Breast Carcinoma" or "Breast Carcinomas" or "Carcinoma, Breast" or "Carcinomas, Breast") |
| #2 | TS=(“Mastectomy, Segmental” or “Mastectomies, Segmental” or “Segmental Mastectomies” or “Segmental Mastectomy” or “Local Excision Mastectomy” or “Local Excision Mastectomies” or “Mastectomies, Local Excision” or “Mastectomy, Local Excision” or Segmentectomy or Segmentectomies or “Partial Mastectomy” or “Mastectomies, Partial” or “Mastectomy, Partial” or “Partial Mastectomies” or “Limited Resection Mastectomy” or “Limited Resection Mastectomies” or “Mastectomies, Limited Resection” or “Mastectomy, Limited Resection” or Lumpectomy or Lumpectomies or “Breast-Conserving Surgery” or “Breast Conserving Surgery” or “Breast Quadrantectomy” or “Breast Quadrantectomies” or “Quadrantectomies, Breast” or “Quadrantectomy, Breast” or “Surgery, Breast-Conserving” or “Breast-Conserving Surgeries” or “Surgeries, Breast-Conserving” or “Surgery, Breast Conserving” or “Breast Conservation Therapy” or “Breast Conservation Therapies” or “Conservation Therapies, Breast” or “Conservation Therapy, Breast” or “Breast-Sparing Surgery” or “Breast Sparing Surgery” or “Breast-Sparing Surgeries” or “Surgeries, Breast-Sparing” or “Surgery, Breast-Sparing”) |
| #3 | #1 AND #2 |
